# Survey Research on Health Inequalities: Exploring the Availability of Indicators of Multiple Forms of Capital in Canadian Datasets

**DOI:** 10.3389/ijph.2021.584916

**Published:** 2021-09-20

**Authors:** Jenny Godley, Katrina Fundytus, Cheyanne Stones, Peter Peller, Lindsay McLaren

**Affiliations:** ^1^ Department of Sociology, University of Calgary, Calgary, AB, Canada; ^2^ Department of Community Health Sciences, University of Calgary, Calgary, AB, Canada; ^3^ Faculty of Nursing, University of British Columbia, Vancouver, BC, Canada; ^4^ Library and Cultural Resources, University of Calgary, Calgary, AB, Canada

**Keywords:** capital, socio-economic position, Canada, survey research, quantitative

## Abstract

**Objective:** Much of the extensive quantitative research linking socio-economic position (SEP) and health utilizes three common indicators: income, occupation and education. Existing survey data may enable researchers to include indicators of additional forms of capital in their analyses, permitting more nuanced consideration of the relationship between SEP and health. Our objective was to identify the breadth of survey questions related to economic, cultural, and social capital available through Statistics Canada surveys, and the extent to which those surveys also include health measures.

**Methods:** We compiled a list of all population-based Statistics Canada surveys, and developed a broad list of potential indicators of forms of capital. We systematically searched the surveys for those indicators and health measures, analyzing their co-occurrence.

**Results:** Traditional SEP indicators were present in 73% of surveys containing health measures, while additional indicators of social and cultural capital were available in 57%.

**Conclusion:** Existing national survey data represent an under-exploited opportunity for research examining the relationship between various forms of capital and health in Canada. Future empirical explorations of these data could enrich our theoretical understanding of health inequities.

## Introduction

Social inequalities in health refer to differences in health which arise from avoidable or unfair social, economic, and environmental conditions [1]. Health is distributed along a gradient where individuals in lower socio-economic positions (SEP) are more likely to suffer a higher disease burden and earlier mortality than those in higher positions [2]. The inverse relationship between SEP and health is well established [3, 4] including in Canada, the geographic focus of the present work [5–10].

Social class has long been considered a fundamental source of social inequality, yet there are many, varied sociological theories of class [11–13]. Recent work in sociology moves beyond the economic conception of social class to focus on cultural and relational aspects. For example, Bourdieu famously argued that social class reflects three overlapping dimensions of capital: economic, cultural, and social [14]. While economic capital includes income and wealth, cultural capital refers to educational attainment, dispositions and *habitus*, and cultural tastes and practices, and social capital refers to social relationships, including networks and the resources embedded within them. Although there has been much work in the sociology of education to try to operationalize these forms of capital in quantitative surveys, particularly the objectified, embodied, and institutional forms of cultural capital, such work is rare in health research [15].

One prominent characteristic of the existing evidence base on social inequalities in health in Canada is that much of the quantitative work is based on a narrow definition of SEP, operationalized through traditional individual-level indicators. Although these indicators certainly capture some of the material and behavioural factors through which social class affects health, they do not capture additional cultural and relational mechanisms that may be at work [16, 17]. To strengthen our understanding of the mechanisms through which SEP affects health, it is useful to consider ways to empirically mobilize broader theoretical conceptualizations of class to include multiple and overlapping economic, social, and cultural forms of capital and corresponding indicators.

### Indicators of SEP

Most quantitative work on social inequalities in health relies heavily (and often solely) on measures of income, occupation, and education to operationalize SEP, both because those are the most common measures available in national-level datasets, and because they reflect traditional theories of how social class operates to affect health (through material and behavioural influences) [6, 8, 9, 18]. These indicators, although important, limit us in terms of our ability to theorize about additional mechanisms through which social class might operate. Recent work has accordingly begun to include additional cultural and relational aspects of SEP [19].

Following the work of Bourdieu [14], several authors elaborate on the notion that “capital” extends beyond economic assets [13, 20, 21]. Health researchers have begun to attempt to operationalize social class more fully by including responses to survey or structured interview questions on cultural and relational measures. In addition to the common measure of educational attainment, cultural capital (or dispositions and habitus) may be measured using questions on various forms of leisure participation, types of media usage, fashion preferences, and other forms of consumption tastes and preferences. Social capital as described by Bourdieu may be measured using questions about social contacts and connections as well as access to resources provided by these relationships [13, 20–22]. Others have included measures of trust, political involvement, and community participation, which operationalize social capital as a community resource, as suggested by Coleman [23].

There are several examples of articles in the *International Journal of Public Health* where authors have moved beyond the traditional measures of income, occupation, and education and incorporated measures of other forms of capital to understand the effects of SEP on health. Frie and Janssen [24] utilized interview data to assess the relationship between latent lifestyle dimensions and health behaviours and outcomes. Villalonga-Olives et al [25] examined the effect of both individual- and group-level social capital on the health status of the elderly in the United States, finding race and ethnic differences. A Dutch study examined the link between “highbrow” cultural participation and health, finding that measures of “distinction” were associated with healthy diet and physical activity, net of education and income [26]. In other international publications, authors have used survey data creatively to operationalize cultural capital beyond educational attainment (such as various forms of leisure participation, types of media usage, fashion preferences, other forms of consumption tastes and preferences) and social capital (social contacts and connections as well as access to resources provided by these relationships) [21, 22].

The Canadian studies that have expanded on traditional measures of SEP have, for the most part, been based on either qualitative or smaller scale survey data and are not nationally representative [27, 28]. One important source for quantitative research on SEP and health is population health surveys administered by national statistical agencies. However, whether, the extent to which, and in what ways, these sources permit researchers to employ a more contemporary theoretical understanding of social class and explore the effects of overlapping forms of capital on health is not known.

### Study Objective

Our objective was to identify the breadth of survey questions related to economic, cultural, and social capital available through Statistics Canada surveys, and the extent to which those surveys also include health measures. The co-occurrence of these questions would suggest that it is possible to conduct theoretically-rich quantitative research on overlapping forms of capital and health using these existing datasets.

## Methods

We focused on national surveys administered by Statistics Canada that are readily available to Canadian university-based academic researchers. Statistics Canada is a federal government agency tasked with collecting and analyzing statistical information pertaining to Canadian culture, resources, economy, and society to various organizations, institutions, and working professionals in Canada [29].

Drawing from the University of Calgary’s Spatial and Numeric Data Services, Data and Statistics website (https://library.ucalgary.ca/sands), we compiled a comprehensive list of nationally representative health and social surveys during the summer of 2017. The initial list included all surveys for which microdata were available to the University of Calgary community, through various sources^1^.

From the full list, we included surveys with the following parameters: 1) a target population that includes adults of all ages (as opposed to, for example, a survey focused only on seniors); 2) population-based (as opposed to individuals within a particular institution, such as a university); and 3) national scope (as opposed to, for example, surveys focused on a single province). The unit of analysis for all included surveys is the individual or household (surveys that gathered data on companies or businesses were excluded). For surveys with multiple cycles or iterations, we only examined the most recent iteration for which we could access documentation.

The survey microdata document files available to Canadian researchers come in two forms: the public use microdata file (PUMF) version (where some potentially identifying variables are suppressed, capped, or aggregated); and the master file version [30]. We were able to access microdata documentation from the master file for 36 surveys; for the remaining 66, we analyzed the microdata documentation from the PUMF files. As we did not require access to survey data, but rather survey documentation, research ethics board approval was not required.

**FIGURE 1:**
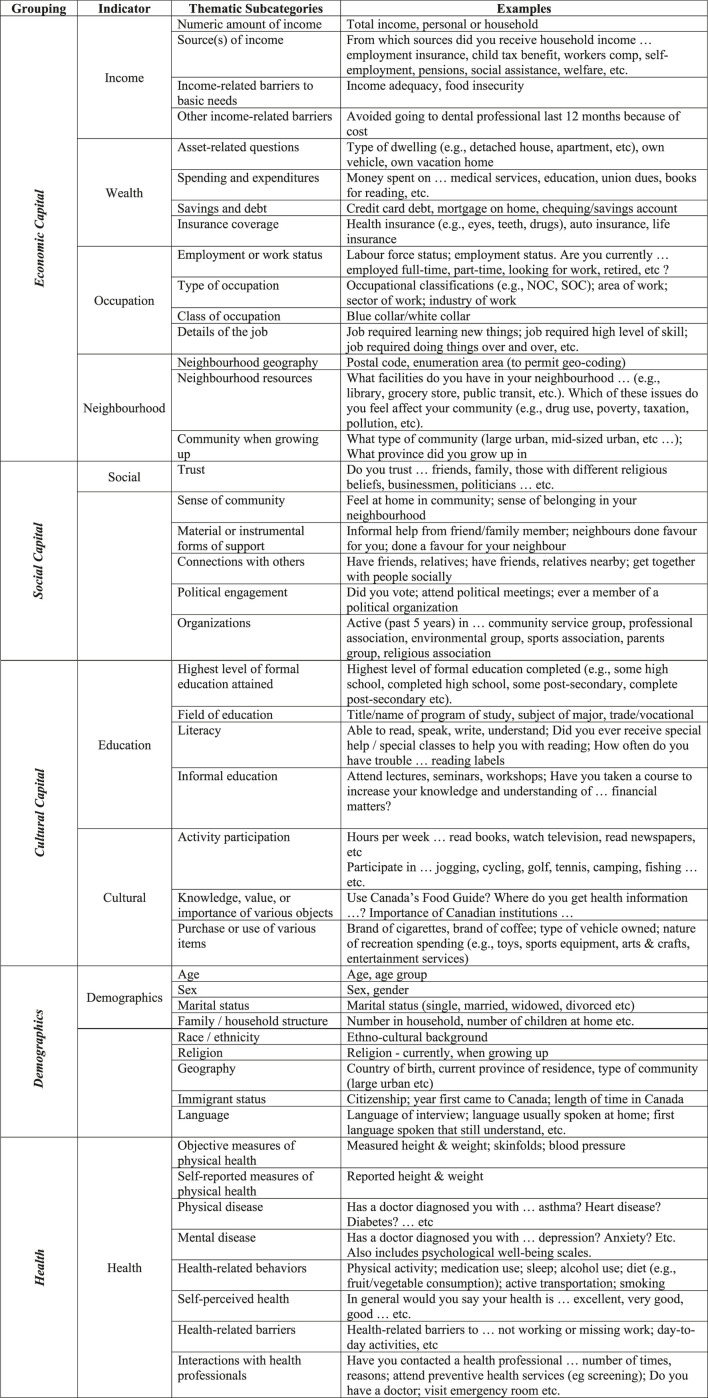
Indicator groupings and thematic subcategories; Forms of Capital in Statistics Canada Surveys Study, 2021.

We used a data extraction template (Figure 1), which we developed and refined in an iterative manner. Three co-authors worked independently to create the template, organizing the indicators into the following thematic areas: economic capital (including income, wealth, and occupation); cultural capital (including education); social capital; self-reported social class; health; and demographic variables (e.g., gender, ethnicity). Following Bourdieu [14], we classified questions that asked about consumption patterns, lifestyle, tastes and preferences as indicators of cultural capital. Following both Bourdieu [14] and Coleman [23], we classified questions that asked about social networks and resources embedded within social networks and trust and civic and community participation, as well as political involvement and participation in organizations, as indicators of social capital.

### Analysis

To illustrate the breadth of survey content on social inequalities and health, we first conducted a qualitative synthesis, where we grouped together similar survey questions to identify sub-categories of each indicator. Second, we quantified the co-occurrence of different forms of SEP indicators and health indicators in the same surveys. To do so, we coded each indicator as present (“1,” at least one measure) or absent (“0,” no measures) in each survey. The data are available as a supplementary file. We examined the percentage of all surveys that had measures of each indicator of SEP, and then each indicator of health.

Not surprisingly, we determined that the traditional measures of income, level of education and occupation were present in a very high proportion (70%) of surveys. Therefore, we started with the co-occurrence of income, occupation and level of education as our baseline SEP measures and then added other indicators one at a time. We systematically examined the co-occurrence of each of those iterations of SEP measures with each of the health measures.

## Results

### Breadth of Indicators

Our initial list contained 194 distinct surveys. After applying our inclusion criteria (adults of all ages; population-based; and national scope), there were 105 distinct surveys (which included a total of 492 survey cycles); however, we were not able to access the survey documentation for three of the surveys. Ultimately, we examined the documentation from a total of 102 surveys (see Supplementary Datasheet S1). The survey dates ranged from 1969 to 2016.

Below, we summarize the breadth of survey content we found, organized using the broad categories in our extraction template (Figure 1). Under each of the broad categories, we classified our indicator questions into thematic sub-categories.

### Economic Capital

Questions about income and occupation at the individual and household levels were very common, but they varied in the level of detail (see Figure 1). Survey questions relating to wealth, which were also common, were divided into four thematic sub-categories. These included: asset-related questions (e.g., ownership of …); questions about spending and expenditures (including broad consumption habits); questions about household saving habits and debt; and questions about insurance coverage or ownership.

Survey questions about neighbourhood-level economic capital included questions about both neighbourhood geography, such as postal code or census divisions and subdivisions, and neighbourhood resources, such as the availability of various services and items in the immediate neighbourhood. Note that questions asking about sense of community in the neighbourhood were classified under Social Capital (below).

### Cultural Capital

The most common question regarding cultural capital was education, which was included in most surveys. Additional cultural capital questions were diverse in nature. Many surveys contained questions around sport, leisure, or physical activity participation, as well as the frequency of involvement. In addition to general questions about activity participation, we observed questions about the specific details or nuances surrounding activity participation, such as personal preferences for how or where leisure time is spent, as well as preferred types or brands of various items.

Another dimension of cultural capital included questions about the knowledge, value, or importance of various objects, such as the importance placed by respondents on arts and cultural institutions and knowledge of guidelines around food and exercise. Finally, many surveys contained questions about the purchase, use or ownership of various items ranging from food expenditures in different types of establishments to owning a boat for recreational purposes.

### Social Capital

Questions about social capital varied in detail and content across the different surveys, and were therefore perhaps the most difficult indicator to identify and classify. Following several iterations, we devised six sub-categories for indicators of social capital. Several surveys had questions on trust, either of the government or of other people. Many surveys asked respondents whether they felt a sense of community or a sense of belonging in their neighbourhood or local area. We also observed survey questions about informational, material or instrumental forms of support from others. We included questions about the size and composition of one’s social network in this latter sub-category.

Some surveys had questions about participating in activities with others, such as exercising and visiting. Questions about political engagement usually took the form of asking about voting behaviour, or interest in politics. And finally, in the social capital category there were questions about membership in organizations, such as churches or volunteer organizations.

### Demographic Questions

All surveys analyzed (*n* = 102) included other demographic questions, which may be related to other aspects of social inequality. Most commonly, surveys included questions about respondent age, as well as gender or sex, and marital status. Some surveys considered family or household structure, such as the number of generations living in the respondent’s household. Another category included those asking about the respondent’s race, ethnicity or religion. Surveys also included geography-related intersections, such as province of residence of birth, census metropolitan area, and rural or urban status. Finally, there were questions about immigrant status and language usage.

### Health

Health indicator questions were common, and ranged from robust and objective measures of health to measures that captured self-reported health-seeking behaviors and perceptions. We classified the health variables into eight categories. Objective physical measures of health refer to clinical measures such as BMI or blood pressure measures. The 1986 Canadian Heart Health Survey and the 2012 Canadian Health Measures Survey (CHMS) had the most comprehensive list of objective physical measures; examples include measured height, weight, blood pressure, and grip strength. Self-reported physical measures of health refer to items such as self-reported (vs. measured) height and weight, which are then used to calculate BMI.

Then there were questions about physical disease such as allergies and chronic conditions. Sometimes these questions are framed in terms of symptoms (e.g., do you have trouble seeing, hearing, walking etc.) and other times they are framed in terms of medical diagnoses (e.g., has a medical professional ever diagnosed you with cancer). Questions about mental disease follow the same pattern, with questions about symptoms (for example, indicators of depression) and diagnoses (have you ever been diagnosed with a mood disorder?).

Several surveys contain questions regarding self-perceived health. A common version asks respondents to rate their health (either physical, mental, or both) on a scale of very poor to excellent. Another sub-category asked respondents about specific health-related behaviours or lifestyle habits, such as drug and alcohol use, smoking behaviours, food consumption, and patterns of physical activity.

A common health indicator question asked in a number of surveys was about health-related barriers or reasons for not participating in or accomplishing various tasks or activities. Most often, questions asked if an illness or disability was the reason for time lost at work or for leaving a job. Some surveys asked about financial difficulties due to long-term disability. A final subcategory included questions that referred to interactions with health professionals or the health care system. Most commonly, these questions asked about health care professional visits, such as going to a doctor or dentist, or overnight hospital stays.

### Frequency and Co-Occurrence of Indicators

#### Frequency of SEP Indicators

Table 1 shows the frequency with which we found each type of indicator, with detailed sub-category data. Income, occupation, and education were the most common indicators of SEP, with all three being available in 70% of all surveys. With respect to additional measures of forms of capital, 54% had at least one additional measure of cultural capital (in addition to education), and 61% of the surveys had at least one additional measure of social capital. Over 50% of the surveys had six or more indicators of SEP, and 50% had measures of income, education, occupation and both social and cultural capital. Perhaps not surprisingly, the only survey that had all eight categories of indicators was the 1983 Class Structure and Class Consciousness Canada Survey.

**TABLE 1:** Number of all surveys (N = 102) in which the indicator category or sub-category is present; Forms of Capital in Statistics Canada Surveys Study, 2021.

Indicator	Number
Economic capital	
Income – any	93
Amount/source	89
Basic needs	24
Barriers	35
Wealth – any	77
Assets	58
Spending	49
Savings/debt	10
Insurance	13
Occupation – any	98
Work status	96
Occupation	78
Class	5
Job details	47
Neighbourhood – any	48
Geography	30
Resources	30
Social capital	
Social capital – any	79
Trust	14
Community	14
Support	57
Others	36
Political	14
Organizations	36
Cultural capital	
Education – any	97
Level	97
Field	18
Literacy	5
Informal	9
Other cultural capital - any	76
Activities	59
Knowledge	21
Purchases	28
Intersections – any	102
Age	100
Sex	100
Marital status	90
Family structure	95
Race/ethnicity	53
Religion	25
Geography	99
Immigration	63
Language	73
Health – any	81
Measured	9
Self-reported measures	13
Physical disease	41
Mental disease	11
Behaviours	36
Self-perceived health	49
Barriers	56
Professionals	40

#### Frequency of Health Variables

A full 73% of the surveys contained at least one health measure. Health items that involved actual physical measurement were the least common, and only appeared in nine percent of the surveys. Self-reported physical measures appeared in thirteen percent of the surveys. Self-reported measures of physical disease were included in 41% of the surveys, while self-reported measures of mental disease were only included in 11% of the surveys. Questions on health-related behaviours were included in 36% of the surveys and questions on interactions with health professionals were included in 40% of the surveys. A question on self-perceived health was asked in almost half (49%) of the surveys. Five surveys contained questions that captured seven of the eight health indicators.

#### Co-Occurrence of Forms of Capital and Health

Table 2 shows the number and percent of surveys with the different types of health questions that also have the various indicators of SEP (column percentages). We start with the common measures of SEP used by many health researchers, namely income, occupation and level of education. Seventy-three percent of the surveys with at least one health variable also include measures of income, occupation, and education. Adding in the additional measures of capital, we see that 66% of the surveys that contain health variables contain measures of income, occupation, education and social capital, and 57% contain measures of income, occupation, education, and additional measures of cultural capital. Forty-five surveys, almost 60% of all the surveys that contain health variables, have measures of both social and cultural capital, in addition to the traditional measures of SEP.

**TABLE 2:** Co-occurrence of social class measures and health measures in the Canadian national surveys (*n* = 102) analyzed; Forms of capital in Statistics Canada Surveys Study, 2021.

		Any health	Measured	Self-reported	Physical dis	Mental dis	Health behav	Self-perceived	Barriers	Professionals
	102	79	9	13	41	11	36	49	56	40
**Income, Occupation, Education**										
N	71	58	7	12	32	8	27	37	41	31
Col. %	70	73	78	92	78	73	75	76	73	78
**Income, Occupation, Educ, and Social capital**										
N	62	52	6	11	29	6	26	35	36	29
Col. %	61	66	67	85	71	55	72	71	64	73
**Income, Occupation, Educ, and Cultural capital**										
N	55	45	7	10	26	6	25	32	29	28
Col. %	54	57	78	77	63	55	69	65	52	70
**Income Occupation, Educ, and BOTH capitals**										
N	51	45	6	9	24	5	24	30	28	26
Col. %	50	57	67	69	59	45	67	61	50	65

## Discussion

Traditional indicators of SEP, including income, occupation, and level of education were quite common amongst our sample of Canadian population-level surveys. These surveys lend themselves to analyses which focus on the economic effects of social class on health. Additionally, there were many interesting and diverse examples of questions pertaining to social and cultural capital, which enable researchers to operationalize additional forms of capital included in Bourdieusian and other theories. We hope that our efforts at classification in Figure 1 will encourage survey researchers in other countries to think broadly about indicators which can be used to operationalize multiple economic, social, and cultural forms of capital from contemporary theoretical perspectives. Examples include health insurance as an indicator of wealth, participating in activities with friends as an indicator of social capital, and forms of recreational activity as an indicator of cultural capital. We have also provided our data as a supplementary file, so that researchers can easily identify which surveys contain which indicators (Supplementary Datasheet S2).

Our findings also illustrate the wide variety of health-related questions available in Canadian national survey data, with objectively measured health variables being much less common than others such as self-perceived health. Some of the health variables may be more useful than others. For example, although questions on health-related barriers were relatively common (56% of the surveys), these questions were often only asked of a subset of survey respondents.

Overall, 79% of surveys contain at least one health measure. Most of these surveys also contained measures of income and level of education, and many also included measures of occupation. Thus, it appears that there are many opportunities to examine the independent and joint effects of the three most common indicators of SEP on health. Many Canadian researchers have taken advantage of these datasets, primarily interpreting the effects of social class on health through a material lens (for examples, see [8, 9, 18]).

Many of these surveys also include questions that could be used to assess other forms of social and cultural capital. Measures of social capital are slightly more common than measures of cultural capital, but a full 57% of surveys with at least one health measure contain both measures of social and cultural capital (in addition to the traditional measures of income, occupation and education). It appears possible, therefore, to conduct quantitative analyses of health inequalities in Canada using both the traditional indicators of SEP and indicators of multiple and overlapping forms of social and cultural capital. Such analyses would add depth to our understanding of how social class influences health, and could be framed and interpreted through a Bourdieusian lens.

### Limitations

One limitation of our study was the inaccessibility of master file documentation for certain surveys. However, we do not believe that this constitutes a significant threat to the validity of our findings. A larger limitation was that we only included the most recent version of each survey cycle. Therefore, we were not able to identify trends or changes over time in the types of questions asked in surveys with multiple cycles, or to determine if earlier cycles contained different or novel questions that may have been discarded in later versions. Additionally, we did not control for the length of surveys. Our findings speak to the presence of absence of certain indicators in the surveys, but not to the actual number of questions or percent of questions.

Finally, qualitative analysis of the survey documentation was a complex process. Some questions fit under multiple indicator categories, or none at all, and largely depended on how a survey question was worded. For example, some of the surveys asked about smoking cigarettes in the following ways (with our classification in parentheses): the preferred brand of cigarette (cultural capital); the amount of cigarettes smoked per day (health); the main reason you began to smoke again (friends of family smoke) (social capital); or have you bought or have you tried to buy cigarettes from a store (wealth). Categorizing the data into distinct indicator categories proved to be a complicated process, which we recognize reflects the reality of the complex interrelationships between social inequality and health. We also fully recognize that while we have identified potential indicators of cultural capital, the classification of those indicators into categories (such as “high brow” and “low brow” as used by Bourdieu) is a complicated, context-specific task.

### Conclusion

Overall, we identified a wide breadth of question content in the national Canadian surveys in terms of indicators of SEP (including indicators that could be used to operationalize various aspects of economic, cultural and social capital), demographic variables, and indicators of health (broadly conceived). Across the Canadian surveys, there is imbalance in the co-occurrence of capital and health indicators, depending on the focus of the survey. The surveys with the highest number of health indicators do not generally contain indicators for all of the forms of capital.

National statistics agencies should be encouraged to continue to include diverse indicators of forms of capital in their surveys. In particular, it would be useful to have more questions that operationalize the economic, cultural, and social aspects of SEP in all surveys that contains health questions. The inclusion of a more robust array of SEP indicators will enable researchers to interpret their findings through a more complex understanding of how social class operates, and therefore to better advise policy makers on the mechanisms through which social inequities in health could be reduced.

## Data Availability

Publicly available datasets were analyzed in this study. This data can be found here: https://www.statcan.gc.ca/eng/start.

## Footnotes

1 Sources include: The Data Liberation Initiative (DLI) and the Research Data Program Center (RDC), the SDA Microdata Analysis and Subsetting System at the University of Toronto Data Library Service, the Statistics Canada NESSTAR data portal, or the Ontario Data Documentation, Extraction Service and Infrastructure (ODESI). The sources available at the University of Calgary are representative of all research universities in Canada.
