# Supplementary material for: Survey Research on Health Inequalities: Exploring the Availability of Indicators of Multiple Forms of Capital in Canadian Datasets
Source: Int J Public Health. 2021 Sep 20;66:584916. doi: 10.3389/ijph.2021.584916 (PMC8489296; doi:10.3389/ijph.2021.584916)
Supplement: Supplementary file 1 [file DataSheet2.docx]

**Appendix A: Survey titles and year**

| **Survey Title** | **Survey Year** |
| --- | --- |
| Access and Support to Education and Training Survey | 2008 |
| Adult Education and Training Survey | 1998 |
| Adult Education Survey | 1984 |
| Adult Training Survey ( superseded by AETS) | 1986 |
| Attitudes and behaviours regarding the non-medical use of drugs: Canadian Adults | 1969-70 |
| Attitudes toward civil liberties and the Canadian Charter of Rights | 1987 |
| Canada Fitness Survey | 1981 |
| Canada Health Monitor Surveys | 1998 |
| Canada Health Survey | 1978-79 |
| Canada Survey of Giving, Volunteering and Participating | 2007 |
| Canada's Alcohol and Other Drugs Survey | 1994 |
| Canadian Addiction Survey | 2004 |
| Canadian Alcohol and Drug Use Monitoring Survey | 2012 |
| Canadian Community Health Survey - Annual Component | 2015 |
| Canadian Community Health Survey - Mental Health & Well-Being | 2002 |
| Canadian Community Health Survey - Mental Health | 2012 |
| Canadian Community Health Survey – Nutrition | 2015 |
| Canadian Community Health Survey: Rapid Response | 2016 |
| Canadian Election Surveys | 2011 |
| Canadian Financial Capability Survey | 2014 |
| Canadian Health Measures Survey | 2012-2013 |
| Canadian Heart Health Survey | 1986-1992 |
| Canadian Income Survey | 2015 |
| Canadian Internet Use Survey (preceded by HIUS) | 2012 |
| Canadian National Health Survey | 2016 |
| Canadian Social Fabric Study | 1997 |
| Canadian Survey of Economic Well-being | 2013 |
| Canadian Survey of Experiences with Primary Health Care | 2008 |
| Canadian Tobacco Use Monitoring Surveys (continued by CTADS) | 2012 |
| Canadian Tobacco, Alcohol and Drugs Survey | 2015 |
| Canadian Travel Survey | 2004 |
| Census of Population | 2006 |
| Class Structure and Class Consciousness: Canada Survey | 1983 |
| CRIC Charter of Rights Survey | 2002 |
| Equality, Security and Community | 1999 |
| Ethnic Diversity Survey | 2002 |
| Family Food Expenditure survey | 1992 |
| Family History Survey | 1984 |
| Food Expenditure Survey | 2001 |
| General Social Survey Giving, Volunteering and Participating | 2013 |
| General Social Survey, Access to and Use of Information Communication Technology | 2000 |
| General Social Survey, Caregiving and Care Receiving | 2012 |
| General Social Survey, Education, Work and Retirement | 1989 |
| General Social Survey, Family | 2006 |
| General Social Survey, Health | 1991 |
| General Social Survey, Social Identity/Social Networks | 2013 |
| General Social Survey, Time Use | 2010 |
| General Social Survey, Victimization | 2014 |
| Health Promotion Survey | 1985 |
| Health Services Access Survey | 2001 |
| Heart Health in Canada | 1998 |
| Household Energy Use Survey | 1993 |
| Household Internet Use Survey (superseded by CIUS) | 2003 |
| Household Facilities by Income and Other Characteristics (also known as Household Facilities and Equipment Survey) | 1997 |
| Households and the Environment Survey | 2015 |
| Individuals Aged 15 years and Over with and without Income | 1998 |
| International Adult Literacy Survey | 1994 |
| International Adult Literacy and Skills Survey - Canada file | 2003 |
| International Survey of Reading Skills | 2004-2005 |
| Joint Canada/United States Survey of Health | 2002-2003 |
| Labour Force Survey | 2012 |
| Labour Market Activity Survey | 1986-1990 |
| Leisure Time Activities and Reading Habits | 1978 |
| Longitudinal and International Study of Adults | 2014 |
| Multiculturalism Attitude Survey | 1991 |
| National Alcohol and Drug Survey | 1989 |
| National Electronic Media Use Survey | 1996 |
| National Household Survey | 2011 |
| National Population Health Survey: Household Component, Cross-sectional | 1998-1999 |
| National Population Health Survey: Household Component, Longitudinal | 2010-2011 |
| National Private Vehicle Use Survey | 1996 |
| National Survey of Giving, Volunteering and Participating | 2000 |
| National Survey on Drinking and Driving | 1988 |
| Nutrition Canada National Survey | 1970-1972 |
| Political Support in Canada | 1993 |
| Program for the International Assessment of Adult Competencies | 2012 |
| Residential Telephone Services Survey | 2013 |
| Retirement and Pre-Retirement Survey | 1975 |
| Smoking Habits of Canadians (also called Survey of Smoking Habits) | 1986 |
| Sun Exposure Survey | 1996 |
| Survey of Annual Work Patterns | 1985 |
| Survey of Canadian Attitudes toward Learning | 2008 |
| Survey of Consumer Finances | 1998 |
| Survey of Emergency Preparedness and Resilience in Canada | 2014 |
| Survey of Family Expenditures | 1969 |
| Survey of Financial Security | 2005 |
| Survey of Fitness, Physical Recreation and Sport | 1976 |
| Survey of Household Energy Use | 2003 |
| Survey of Household Spending | 2009 |
| Survey of Labour and Income Dynamics | 2011 |
| Survey of Literacy Skills Used in Daily Activities | 1989 |
| Survey of 1981 Work History | 1981 |
| Survey of Reading and Buying Books | 2005 |
| Survey of Selected Leisure Time Activities | 1975 |
| Survey of Volunteer Activity | 1987 |
| Survey of Volunteer Workers | 1979-80 |
| Survey of Voters and Non-Voters | 2002 |
| Survey on Smoking in Canada | 1994-1995 |
| Survey on the Importance of Wildlife/Nature to Canadians | 1996 |
| Tourism Attitude and Motivation Study | 1983 |
| Travel Activities and Motivation Survey | 2006 |
| Travel Survey of Residents of Canada | 2015 |
